# The crystal structure of haemoglobin from Atlantic cod

**DOI:** 10.1107/S2053230X1900904X

**Published:** 2019-07-16

**Authors:** Ronny Helland, Eva Katrin Bjørkeng, Ulli Rothweiler, Magne Olav Sydnes, Daniela Maria Pampanin

**Affiliations:** a NorStruct, Department of Chemistry, Faculty of Science and Technology, UiT – The Arctic University of Norway, NO-9037 Tromsø, Norway; b Department of Chemistry, Bioscience and Environmental Engineering, University of Stavanger, Faculty of Science and Technology, NO-4036 Stavanger, Norway; c NORCE AS, Prof. Olav Hanssensvei 15, NO-4021 Stavanger, Norway

**Keywords:** haemoglobin, *Gadus morhua*, Atlantic cod

## Abstract

The crystal structure of haemoglobin from Atlantic cod has been solved to 2.54 Å resolution. The structure consists of two tetramers in the crystallographic asymmetric unit. The structure of haemoglobin obtained from one individual cod suggests polymorphism in the tetrameric assembly.

## Abstract 2

The isolation of the dominant isoform of haemoglobin from Atlantic cod caught in southwest Norway is reported and its X-ray crystal structure is presented.

## 1. Introduction   

Increasing the knowledge of how Atlantic cod (*Gadus morhua*) is affected by chemicals that contaminate the aquatic environment has been a longstanding effort (Karlsen *et al.*, 2011 ▸; Pampanin, 2017 ▸; Pampanin *et al.*, 2014 ▸, 2016 ▸; Pampanin & Sydnes, 2013 ▸; Sundt *et al.*, 2012 ▸). Environmental pollutants such as polycyclic aromatic hydrocarbons (PAHs), resulting for example from oil exploration and production operations or accidental oil spills, have received attention from researchers since the 1970s owing to their carcinogenic potential (United States Environmental Protection Agency, 2009 ▸). However, only in recent years, with the development of new and more sensitive analytical methods, has it become possible to obtain a deeper understanding of the adverse effects and the mode of action of these contaminants (Beyer *et al.*, 2011 ▸; Enerstvedt, Sydnes, Larssen *et al.*, 2018 ▸; Enerstvedt *et al.*, 2017 ▸; Enerstvedt, Sydnes & Pampanin, 2018 ▸; Karlsen *et al.*, 2011 ▸).

In fish, PAHs are metabolized *in vivo* in order to make them more water-soluble, thus facilitating excretion (Pampanin & Sydnes, 2013 ▸). However, this process generates products that can potentially be more toxic than the parent compounds. Some of them can react with molecules such as DNA and proteins and form adducts, producing genotoxic effects or impairment of metabolism (Pampanin *et al.*, 2017 ▸). In humans, adducts of haemoglobin have been studied substantially as an important method of evaluating individual health conditions (Stedingk *et al.*, 2012 ▸). As an example, PAHs derived from tobacco smoking have been found to make adducts with particular amino acids in haemoglobin (Phillips & Venitt, 2012 ▸). It is reasonable to believe that this would also be the case for Atlantic cod, and this hypothesis is strengthened by the fact that PAH adducts with other blood proteins have been identified in this fish species (Enerstvedt *et al.*, 2017 ▸; Enerstvedt, Sydnes & Pampanin, 2018 ▸). The potential use of haemoglobin adducts as potential biomarkers of PAH exposure in Atlantic cod is under investigation in our research group. Therefore, in order to study the structural effects that a PAH adduct would have on haemoglobin, the structure of this protein has to be defined in the presence and the absence of the adduct. Firstly, however, pure haemoglobin needs to be obtained. The full genome of Atlantic cod has been sequenced (Star *et al.*, 2011 ▸), and about 70 isoforms of haemoglobin have been proposed by the EST translation of the genome. However, sequence similarity within the α and β forms of cod haemoglobin is high, simplifying the structure-identification research challenge. Here, isolation of the dominant isoform of haemoglobin from Atlantic cod caught in southwest Norway is reported and its X-ray crystal structure is presented.

## 2. Materials and methods   

### 2.1. Macromolecule production   

#### 2.1.1. Blood sampling   

An individual Atlantic cod caught in Idsefjord, Stavanger, Norway using baited traps was kept in a 1000 l glass-fibre tank with a continuous-flow system (water temperature 8°C, salinity 33‰, light/darkness 8/16 h, water flow 7–9 l min^−1^). Haemoglobin was obtained from the fish by extracting blood samples over a period of four weeks (total amount 10 ml). Heparin was used as an anticoagulant and the blood samples were centrifuged for 5 min at 2000*g* and 4°C. Plasma fractions were removed and the remaining red blood cell (RBC) fractions were kept at −80°C until further analysis.

#### 2.1.2. Purification   

**Figure 1:**
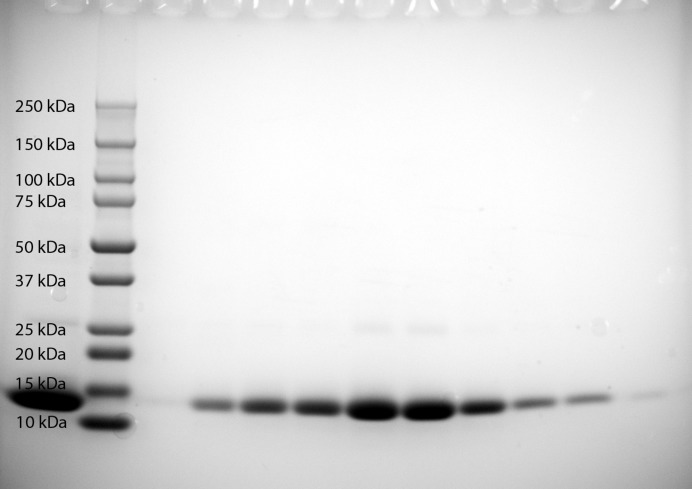
SDS–PAGE illustrating the purity of fractions containing cod haemoglobin.

A blood sample of about 500 µl from the cod was thawed and lysed by incubating the sample overnight at 4°C in 8 ml 4 m*M* Tris pH 7.5, 10 m*M* NaCl, 2 µl DNaseI. 2 ml of the lysed sample was diluted to 10 ml using 50 m*M* Tris pH 9.5 (buffer *A*). Samples were subsequently applied onto a 5 ml HiTrap QHP column (IEX) for anion-exchange chromatography. A washing step using 60% 50 m*M* bis-Tris propane pH 5.5 (buffer *B*) was included before eluting the protein using a gradient from 60% to 100% buffer *B* over 60 ml. Fractions containing haemoglobin were subjected to sodium dodecyl sulfate–polyacrylamide gel electrophoresis (SDS–PAGE) for assessment of purity (Fig. 1 ▸) before being pooled and concentrated to 16 mg ml^−1^ using 3K Amicon Ultra spin filters.

### 2.2. Crystallization   

**Figure 2:**
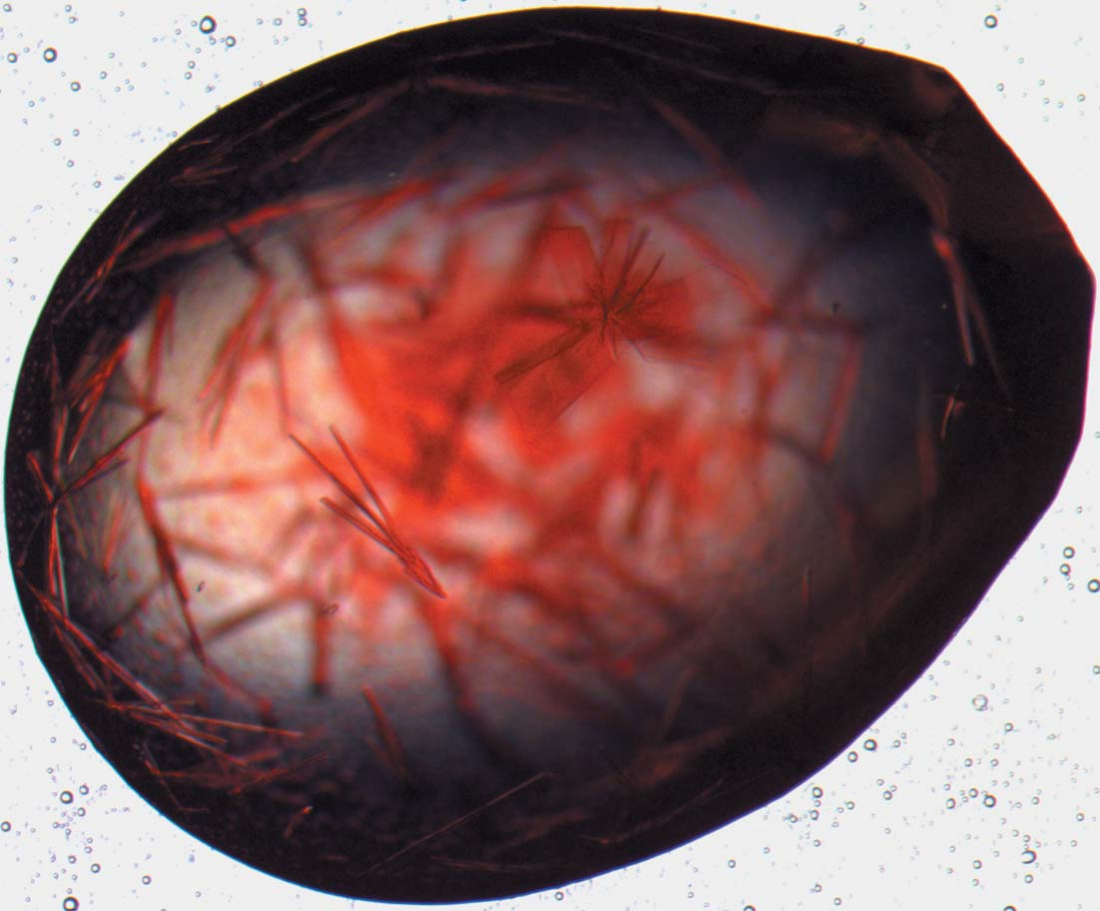
Cod haemoglobin crystals.

Initial crystallization conditions were screened with a Phoenix crystallization robot (Art Robbins Instruments) using the sitting-drop vapour-diffusion method. A total of 480 in-house conditions were screened in MRC plates with 60 µl reservoir solution per well; drop solutions were prepared by mixing 0.25 µl well solution and 0.25 µl protein solution at 16 mg ml^−1^. Further optimization in Hampton Research 24-well hanging-drop plates using 500 µl reservoirs, a protein concentration of 8 mg ml^−1^ and 1 µl + 1 µl drops yielded thin needle-shaped and plate-like crystals from 12% PEG 5K MME, 0.1 *M* Bicine pH 8.5 (Table 1 ▸, Fig. 2 ▸).

### 2.3. Data collection, processing and structure determination   

Data were collected on beamline ID23-1 at the ESRF (Nurizzo *et al.*, 2006 ▸). All data were integrated and scaled using *XDS* (Kabsch, 2010 ▸) and *AIMLESS* (Evans & Murshudov, 2013 ▸; Winn *et al.*, 2011 ▸). Data-collection statistics are listed in Table 2 ▸. The structure was solved by molecular replacement in *Phaser* (McCoy *et al.*, 2007 ▸) and subsequent autobuilding using *RESOLVE* in *PHENIX* (Adams *et al.*, 2010 ▸; Afonine *et al.*, 2012 ▸; Grosse-Kunstleve & Adams, 2003 ▸; Moriarty *et al.*, 2009 ▸; Terwilliger, 2004 ▸; Terwilliger *et al.*, 2008 ▸, 2009 ▸; Zwart, 2005 ▸).

The template for molecular replacement was generated by homology modelling using the structure of haemoglobin from yellow perch (PDB entry 1xq5; Center for Eukaryotic Structural Genomics, unpublished work) and *BLAST* sequences for the α and β chains of cod haemoglobin (GenBank codes ABV21458.1 and ACV69847.1, respectively). Homology models of the α and β chains were generated using the Schrödinger software suite (https://www.schrodinger.com/) and were assembled into a tetramer similar to PDB entry 1xq5.

Inspection of electron-density maps was carried out in *Coot* (Emsley *et al.*, 2010 ▸), followed by restrained positional refinement in *REFMAC* (Murshudov *et al.*, 2011 ▸). Refinement statistics are listed in Table 3 ▸. To confirm the polypeptide sequence, cod haemoglobin was subjected to in-solution digestion and subsequent mass-spectrometric analysis.

The structure of cod haemoglobin has been deposited in the Protein Data Bank as entry 6hit.

## 3. Results and discussion   

A new purification protocol was established by modifying the method described previously by Delatorre *et al.* (2000 ▸). The sample was loaded onto an anion-exchange chromatography column at high pH and was eluted by a gradual reduction of the pH. Cod haemoglobin at 16 mg ml^−1^ with a purity sufficient for crystallographic studies was obtained (Fig. 1 ▸).

Thin needle-shaped and plate-like crystals grew in bundles from 12% PEG 5K MME, 0.1 *M* Bicine pH 8.5 (Fig. 2 ▸). Optimization of the crystallization conditions, in an attempt to obtain larger single crystals, proved to be difficult. The optimization included a reduction in the protein and/or precipitant concentration, seeding and the use of additives. Single crystals were difficult to isolate and data could often not be processed because of multiple crystals or lattices. The best data were collected to 2.5 Å resolution from a crystal of approximately 0.3 × 0.05 × 0.05 mm in size on beamline ID23-1 at the ESRF. The crystal belonged to space group *P*2_1_2_1_2_1_, with unit-cell parameters *a* = 62, *b* = 103, *c* = 199 Å. Data-collection statistics are listed in Table 1 ▸.

**Figure 3:**
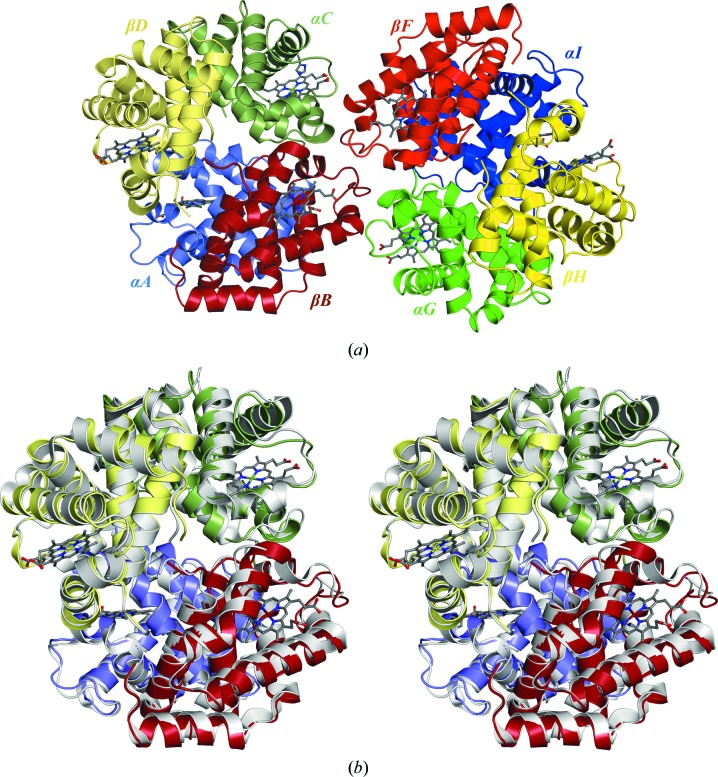
(*a*) Ribbon illustration of the two cod haemoglobin tetramers in the crystallographic asymmetric unit. (*b*) Stereoview showing superimposition of yellow perch haemoglobin (grey) on cod haemoglobin tetramer *ABCD*.

The crystal volume-to-molecular weight ratio suggested the presence of eight haemoglobin molecules in the asymmetric unit. A homology model of tetrameric cod haemoglobin (made for pre-crystallographic studies) was used as a template for structure determination using molecular replacement. *Phaser* (McCoy *et al.*, 2007 ▸) was able to identify two tetramers in the asymmetric unit [Fig. 3 ▸(*a*)]. The solution was confirmed by running *Phaser* using PDB entry 1xq5 as the template. Autobuilding was carried out using the ‘simulated annealing’ option with the tetrameric homology model as the starting model instead of PDB entry 1xq5. The model was chosen to avoid fragmentation of the polypeptide sequences during autobuilding and to maintain the intrinsic order of the α and β chains, which are structurally very similar. The homology-based sequences were edited to correspond to the sequences obtained by mass spectrometry. Manual refitting of electron-density maps and subsequent restrained positional refinement, including isotropic *B*-factor refinement, resulted in a final structure with *R*_work_ = 23.23% and *R*_free_ = 30.13%.

The cod haemoglobin shares 64% and 74% sequence identity with the yellow perch haemoglobin (PDB entry 1xq5) α and β chains, respectively, and the yellow perch tetramer superimposes on that of cod haemoglobin with r.m.s.d.s of 1.02 and 0.96 Å for the *ABCD* and *EFGH* tetramers, respectively [Fig. 3 ▸(*b*)]. The two cod haemoglobin tetramers superimpose on each other with an r.m.s.d. of 0.51 Å. The r.m.s.d.s between individual monomers are of the order of 0.38–0.78 and 0.46–1.12 for the α and β chains, respectively. Differences in conformation are primarily located in the loop between residues 45–60 and the C-terminal loops. Differences in conformation are observed in the same regions between the cod and perch haemoglobins.

The electron density is generally well defined for internal residues, but can be weaker for surface-exposed side chains. The quality of the electron density differs between polypeptide chains that are identical (*i.e.* chains *A*, *C*, *E* and *G* and chains *B*, *D*, *F* and *H*). In places where the electron density is weak, the conformation of the peptide is based on the conformation of other chains in which the electron density is better defined. This could be the explanation for the relatively large difference between *R* and *R*_free_.

**Figure 4:**
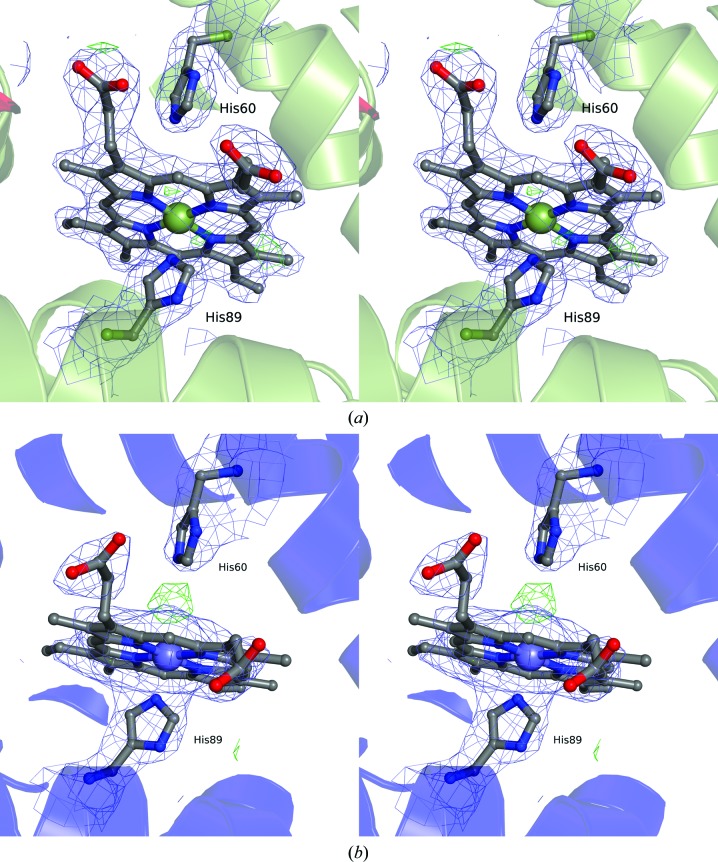
Stereoview illustrating the electron density for heme α-unit chain *C* (*a*) and chain *E* (*b*). 2*F*_o_ − *F*_c_ (blue) and *F*_o_ − *F*_c_ (green) maps are contoured at 1σ and 3σ, respectively.

Eight heme groups could be located in the electron density. The residues forming heme-binding sites are reasonably well defined, as are the heme propionate groups. The heme iron appears to be pentacoordinated in all subunits. Weak difference density in the vicinity of the iron in some of the heme groups may suggest a coordinated water or hydroxyl molecule at the sixth coordinating position, as observed in haemoglobin from, for example, perch (PDB entry 3bj3; Aranda *et al.*, 2009 ▸). However, additional studies are needed to confirm this. The side chains of the histidines in the α and β units have conformations similar to that observed for perch haemoglobin, with distances of about 4.0–4.4 and 2.1–2.3 Å between the histidinyl N^∊2^ atom and Fe for α-chain residues His60 and His89, respectively, and 4 and 2.1–2.4 Å for β-chain residues His64 and His93, respectively. Haemoglobins from cold-adapted species have been found to follow distinct autooxidation patterns (Vitagliano *et al.*, 2004 ▸, 2008 ▸; Aranda *et al.*, 2009 ▸). The small differences in the distances and the amount of difference density observed close to the heme iron may suggest different states of autooxidation (Fig. 4 ▸). The electron density suggests that the tetramers may consist of different isoforms of the α and β subunits. This is not totally unexpected since heterogeneity in fish haemoglobins has been suggested previously (Sick, 1961 ▸; Andersen *et al.*, 2009 ▸). For example, the electron density of α-unit chain *A*, but not chain *C*, resembles the sequence (ACJ66341) reported by Andersen *et al.* (2009 ▸). However, refinement using this sequence did not improve the refinement statistics, and the *R* and *R*_free_ values were about 5% higher. Similarly, the β-unit chain *D* (and *H*) appears to have Ala at position 68 instead of Ile or Val which are usually found at this position. The cod haemoglobin structure presented in this work appears to have Ala at position 63 and Leu at position 56 in the β chains, instead of Lys or Met, which are more common in cod sequences. Work by Andersen *et al.* (2009 ▸) suggests that smaller residues in these positions increase the oxygen affinity.

Blood samples were collected from a single individual fish with the aim of avoiding polymorphism owing to genetic variation. Although the resolution of the data is sufficient to trace most of the polypeptide chains, it is not sufficient to specifically identify particular amino-acid residues. The difference electron-density maps suggest polymorphism in both the α and the β chains. However, the majority of the polypeptide chains could be traced with adequate certainty and can thus serve as a starting point for further analysis of adduct formation in Atlantic cod haemoglobin exposed to PAH contamination.

## Supplementary Material

PDB reference: cod haemoglobin, 6hit

## Figures and Tables

**Table 1:** Crystallization conditions for cod haemoglobin

Method	Hanging drop
Plate type	24-well, Hampton Research
Temperature (K)	298
Protein concentration (mg ml^−1^)	8
Composition of reservoir solution	0.1 *M* Bicine pH 8.5, 12% PEG 5K MME
Volume and ratio of drop	0.25 µl + 0.25 µl
Volume of reservoir (µl)	500

**Table 2:** Data-collection and processing statistics Values in parentheses are for the outer shell.

Diffraction source	ID23-1, ESRF
Wavelength (Å)	0.9840
Temperature (K)	100
Detector	PILATUS 6M-F
Crystal-to-detector distance (mm)	498.92
Rotation range per image (°)	0.15
Total rotation range (°)	120
Space group	*P*2_1_2_1_2_1_
*a*, *b*, *c* (Å)	62.69, 103.26, 199.35
α, β, γ (°)	90, 90, 90
Mosaicity (°)	0.2
Resolution range (Å)	50–2.54 (2.63–2.54)
Total No. of reflections	197786 (18871)
No. of unique reflections	45021 (4302)
Completeness (%)	98.8 (97.3)
Multiplicity	4.4 (4.4)
*I*/σ(*I*)†	7.2 (0.9)
〈*I*/σ(*I*)〉	10.6 (1.7)
*R*_r.i.m._ (%)	10.0 (89.4)
*R*_merge_ (%)	7.9 (71.6)
CC_1/2_	0.997 (0.597)
Overall *B* factor from Wilson plot (Å^2^)	49.2

† *I*/σ(*I*) in the outer shell falls below 2.0 at 2.8 Å.

**Table 3:** Structure-solution and refinement statistics Values in parentheses are for the outer shell.

Resolution range (Å)	50–2.54 (2.56–2.54)
Completeness (%)	98.3 (96.3)
σ Cutoff	None
No. of reflections, working set	40577 (3047)
No. of reflections, test set	2191 (142)
Final *R*_cryst_ (%)	23.23 (32.7)
Final *R*_free_ (%)	30.13 (36.2)
Cruickshank DPI	0.6
No. of non-H atoms	
Protein	8582
Ligand	240
Solvent	0
R.m.s. deviations from ideal values	
Bond lengths (Å)	0.013
Angles (°)	1.607
Average *B* factors (Å^2^)	
Protein	59.8
Ligand	57.9
Ramachandran plot	
Most favoured (%)	90.7
Allowed (%)	8.4
Generously allowed (%)	0.6
Disallowed (%)	0.3
